# Toward Sustainable Testosterone Manufacturing: Green Chemistry and Microbial Biotransformation Approaches

**DOI:** 10.3390/ijms27052444

**Published:** 2026-03-06

**Authors:** José M. Fernández-Cañón, Alejandro Chamizo-Ampudia

**Affiliations:** 1 Área de Bioquímica y Biología Molecular, Departamento de Biología Molecular, Universidad de León, 24007 León, Spain; jmferc@unileon.es; 2 Institute of Molecular Biology, Genomics and Proteomics (INBIOMIC), Universidad de León, Campus de Vegazana, 24071 León, Spain

**Keywords:** androstenedione, cytochrome P450, green chemistry, microbial cell factories, phytosterol metabolism, steroid biotransformation, testosterone production, 17β-hydroxysteroid dehydrogenase

## Abstract

Testosterone is a vital steroid hormone with important physiological roles and broad clinical significance, serving as a central molecular precursor in the synthesis of many pharmacologically active steroids. Testosterone is traditionally produced through complex chemical synthesis routes that involve hazardous reagents, harsh conditions, and produce significant toxic waste. In recent decades, growing regulatory requirements and environmental sustainability goals have spurred the development of alternative biotechnological methods that use microbial biotransformation. This review offers a comparative analysis of chemical and biological methods for producing testosterone, focusing on microbial steroid biotransformation pathways and the key enzymatic steps involved in testosterone biosynthesis. It examines key advances in sterol breakdown, pathway engineering, and enzyme driven modifications, including the roles of 17β-hydroxysteroid dehydrogenases and cytochrome P450 monooxygenases. The performance, specificity, and environmental impacts of bacterial and fungal cells as cell factories, especially *Mycolicibacterium* and *Aspergillus* species, are critically analyzed within the framework of modern green chemistry principles. Overall, by combining molecular insights with process considerations, this review illustrates how microbial platforms could complement and gradually transform traditional chemical synthesis methods, promoting a shift toward more sustainable steroid hormone production through engineered biocatalysts.

## 1. Introduction

Steroid hormones represent a fundamental class of bioactive molecules that exert pleiotropic effects in human physiology and pathology [1]. Characterized by their tetracyclic core structure derived from cholesterol, steroid hormones play a crucial role in regulating inflammation, electrolyte balance, metabolism, immune modulation, sexual differentiation, and reproductive function [2]. These compounds constitute a biochemical cornerstone, serving as precursors and intermediates in the biosynthesis of a broad range of physiologically and pharmacologically relevant steroids, including glucocorticoids, mineralocorticoids, androgens, estrogens, and progestogens [3]. Due to their extensive involvement in essential biological processes, steroid hormones are, alongside antibiotics, considered among the most indispensable pharmaceutical agents developed in the 20th century [4].

Pharmacologically relevant steroids include glucocorticoids such as cortisol and dexamethasone, mineralocorticoids such as aldosterone, androgens such as testosterone and dihydrotestosterone, estrogens such as estradiol, and progestogens such as progesterone. These compounds are extensively used in the treatment of endocrine disorders as such Addison’s disease and hypogonadism, inflammatory and autoimmune diseases as such rheumatoid arthritis and asthma, neoplastic diseases as such breast and prostate cancer, and for contraceptive and hormone replacement purposes [5]. Among these, testosterone (TS) occupies a central role not only as a vital androgenic hormone regulating male sexual development, muscle mass, bone density, and overall metabolic homeostasis, but also as a critical biochemical precursor in the biosynthetic pathways leading to estrogens and other steroid hormones. This dual function underscores TS’s unique pharmacological and industrial importance [6].

Economically, the steroid pharmaceutical market represents a robust and expanding segment of the global healthcare industry. As of 2022, the global market value for steroid drugs was estimated at over USD 3.32 billion, with forecasts projecting growth to USD 17.5 billion by 2030, driven by increased prevalence of chronic diseases, aging populations, and expanding therapeutic indications [7]. Within this context, TS stands out as one of the most in demand steroid hormones worldwide, particularly in the context of TS replacement therapy (TRT), age-associated hypogonadism, sarcopenia, and gender-affirming hormonal treatments for transgender individuals [8].

The industrial production of TS has traditionally relied on multistep chemical synthesis, often starting from plant-derived sterols such as diosgenin or stigmasterol [9]. While effective in terms of yield, these processes commonly involve environmentally hazardous reagents such as chromium-based oxidants, toxic organic solvents such as chloroform and benzene, and high energy consumption, thereby raising concerns about sustainability and environmental safety [10]. In light of these challenges, biotechnological alternatives have gained increasing attention as sustainable and scalable platforms for the production of steroid hormones.

Recent advances in microbial biotechnology have enabled the development of biotransformation systems using genetically engineered microorganisms, including species of *Mycolicibacterium*, *Rhodococcus*, and *Aspergillus*, to convert phytosterols into steroid intermediates through regio- and stereoselective oxidation, hydroxylation, and dehydrogenation reactions [11,12]. Moreover, metabolic engineering and synthetic biology approaches are being applied to optimize these microbial platforms for higher yields, pathway stability, and process integration, aligning with principles of green chemistry and circular bioeconomy [13].

**Figure 1:**
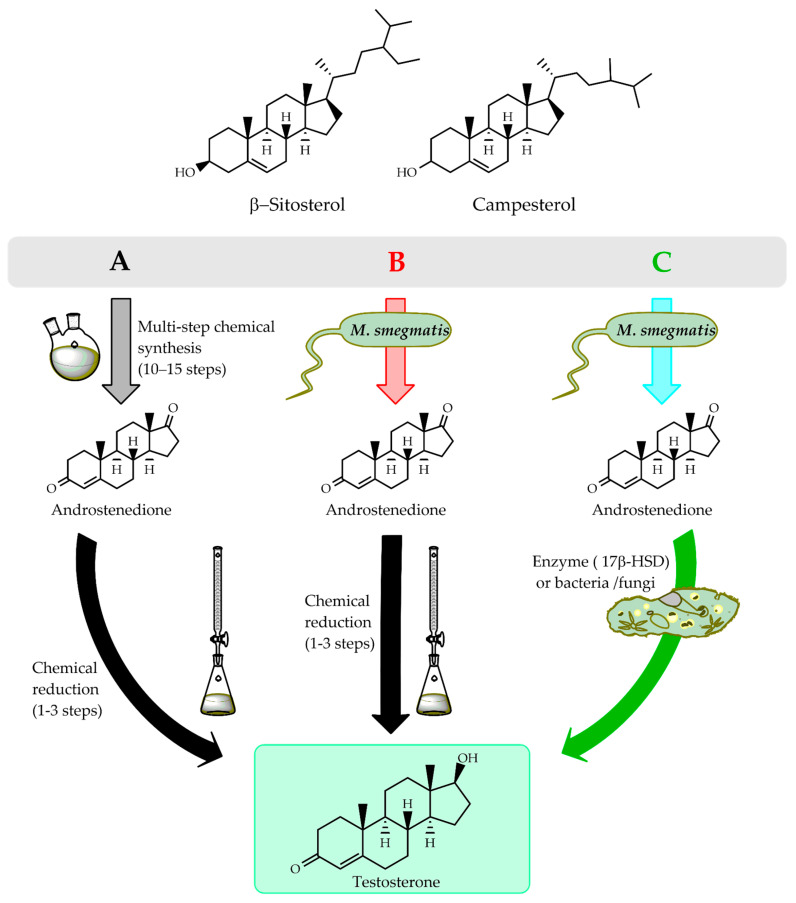
Chemical and biotechnological routes for industrial TS production. Schematic comparison of conventional chemical synthesis (**A**), hybrid chemical–biological processes (**B**), and fully biotechnological routes (**C**) for TS manufacturing. Chemical routes rely on multistep transformations using hazardous reagents and organic solvents, whereas biotechnological approaches integrate microbial sterol biotransformation and enzyme catalyzed reactions under milder conditions. Hybrid routes currently represent the dominant industrial strategy.

The objective of this review is to provide a comprehensive and comparative overview of chemical and biotechnological strategies for TS production, critically evaluating their industrial feasibility, environmental impact, and potential for integration into sustainable production models. This analysis aims to inform future efforts toward cleaner and more efficient manufacturing of steroid hormones within a circular economy framework. An overview of conventional chemical, hybrid, and fully biotechnological routes for industrial TS production is schematically presented in Figure 1.

## 2. Industrial Applications and Market for Testosterone

TS, as the primary androgenic hormone in humans, is recognized for its crucial role in the development and maintenance of male sexual characteristics, anabolic metabolism, mood regulation, and bone health. Its clinical use has been primarily established in hormone replacement therapy for men with primary or secondary hypogonadism, a condition affecting between 2% and 6% of older adult males, with prevalence increasing with age and chronic diseases [14,15].

Beyond hypogonadism, TS has been employed in the prevention and treatment of osteoporosis, especially in men presenting low bone mass associated with androgen deficiency, given its influence on bone remodeling and mineral density [16,17]. The hormone has also been acknowledged for its capacity to improve muscle mass and strength in patients suffering from cachexia or sarcopenia related to illnesses such as HIV, chronic kidney disease, and neuromuscular disorders, demonstrating significant clinical benefits in quality of life and functionality [8,18,19,20].

In the field of female reproductive health, although less prevalent, TS has been applied in the treatment of sexual dysfunctions, such as decreased libido, under strict medical supervision and in low dose formulations [21,22]. This use, highlights the pharmacological versatility of the hormone and opens emerging research avenues aimed at broadening its therapeutic profile.

Concurrently, TS is subject to a strictly regulated parallel market within sports and bodybuilding. Its use outside official medical indications, for athletic performance enhancement and muscle mass gain is prohibited by international bodies such as the World Antidoping Agency (WADA), yet significant demand persists in this sector, creating global regulatory and health challenges [23].

From an economic standpoint, the global TS market constitutes a robust and expanding segment of the pharmaceutical industry. According to data from Fortune Business Insights and recent sector reports, the TS market exceeded USD 2.5 billion in 2023 and is projected to surpass USD 3.5 billion by 2032, with a compound annual growth rate (CAGR) exceeding 5%, driven primarily by demographic aging, increased diagnosis of hormonal disorders, and growing demand for personalized therapies [24,25]. A summary of global market size and projected growth trends for TS is presented in Table 1.

The market is segmented according to administration route (oral, injectable, transdermal, subcutaneous), pharmaceutical formulations (immediate versus controlled release), and geographical regions. Injectable products continue to dominate due to their high bioavailability and lower dosing frequency, although transdermal formulations (gels, patches) and oral forms (esters, undecanoates) have been gaining market share due to convenience and improved patient adherence [26]. Notably, advances in nanotechnology and controlled release delivery systems have revolutionized TS pharmacokinetics, reducing side effects and optimizing the therapeutic profile, thereby opening new opportunities for the industry [27,28]. The relative market distribution of TS products according to the route of administration is summarized in Table 2.

Regulatory considerations classify TS as a controlled substance in multiple countries owing to its potential for abuse and adverse effects, thus production, distribution, and commercialization are subjected to stringent international regulations, such as those enforced by the Food and Drug Administration (FDA) (United States), European Medicines Agency (EMA) (Europe), and other regional health agencies [29]. This rigorous regulatory framework compels the industry to maintain high standards of quality, traceability, and safety, increasing production and technical costs while ensuring patient protection and risk minimization.

The industrial importance of TS also resides in its function as a precursor for the synthesis of other bioactive steroids, both in pharmaceuticals and biotechnology [30]. The efficient and sustainable synthesis of TS directly impacts the availability of derivatives such as dihydrotestosterone, anabolic steroids, and modified progestins, thereby expanding the therapeutic spectrum of the steroid family [31,32].

Finally, within the current framework of circular economy and sustainability, TS production faces the challenge of incorporating greener processes, both in traditional chemical synthesis and emerging biotechnological routes. Growing demand and environmental regulations render the optimization of production processes to minimize environmental impact, energy consumption, and toxic waste generation indispensable [33,34]. These issues are being addressed through microbial metabolic engineering, biocatalysis, and green chemistry applications in the pharmaceutical industry [35,36].

TS is not only regarded as an essential drug with a growing market and diversified clinical applications, but its efficient, sustainable, and safe production is also considered a strategic pillar for the pharmaceutical industry, biotechnology, and global public health.

## 3. Testosterone Chemical Production: Industrial Perspectives

The classical production of TS by chemical means predominantly utilizes cholesterol and phytosterols such as β-sitosterol, stigmasterol, and campesterol, which are extracted from vegetable oils and subsequently subjected to multistep transformations. These include side-chain cleavage, oxidation, reduction, and molecular rearrangements, leading to the production of key intermediates such as AD or dehydroepiandrosterone (DHEA), which are further converted into TS. Processes described in patents such as WO2017093980A1 [37] and US2742485A [38] typically involve 10 to 15 synthetic steps with overall conversion efficiencies ranging between 50% and 75%, depending on the purity of the starting material and optimization of catalytic steps. Representative patented chemical routes for industrial TS synthesis, including starting materials, number of reaction steps, key reagents, and reported yields, are summarized in Table 3.

This table summarizes selected chemical and electrochemical patents describing industrial routes for TS production, highlighting starting materials, key intermediates, number of synthetic steps, main reagents and solvents, reported yields, and associated environmental and safety concerns. The comparison illustrates the complexity, reagent intensity, and waste generation typically associated with conventional chemical synthesis approaches.

Despite the environmental and safety concerns discussed below, classical chemical synthesis routes offer several technical advantages that have historically supported their industrial dominance. These approaches are characterized by high reproducibility, well-defined reaction mechanisms, short production timelines, and compatibility with established large-scale chemical manufacturing infrastructure. Reaction parameters can be precisely controlled, and product profiles are generally predictable, facilitating regulatory validation and process standardization. Such attributes have contributed to the continued reliance on chemical synthesis in commercial testosterone production.

These methods rely on an array of toxic solvents and reagents. Chlorinated solvents such as dichloromethane (DCM) and chloroform are frequently used due to their capacity to dissolve both lipophilic phytosterols and intermediate steroid compounds. However, these solvents are associated with carcinogenicity, hepatotoxicity, and poor biodegradability [44]. Inhalation or dermal exposure during industrial handling increases the risk of systemic toxicity, posing substantial occupational hazards. Similarly, aromatic hydrocarbons like toluene and xylene, often employed during reaction and purification steps, are known neurotoxins with significant environmental persistence [45]. Their use contributes to volatile organic compound (VOC) emissions and ozone formation in the troposphere [46].

Moreover, several oxidation steps in these processes employ reagents such as chromium (VI) salts (potassium dichromate), selenium dioxide, or peracids, which generate heavy metal waste or corrosive byproducts [47,48]. Hexavalent chromium, in particular, is recognized for its carcinogenic potential and its resistance to environmental degradation, prompting strict regulatory restrictions under REACH and EPA guidelines [49,50]. The treatment of these effluents requires energy intensive and costly neutralization and disposal procedures [51].

A significant proportion of the synthetic routes also generates undesirable byproducts. For example, during side-chain cleavage of sitosterol, ketosteroid derivatives and oxidized sterols are produced, many of which exhibit poor aqueous solubility and can act as endocrine disrupting compounds (EDCs) [52]. If not properly managed, these compounds may enter water systems, where they can impair reproductive systems in aquatic organisms, even at concentrations as low as nanomolar levels [53,54]. Persistent detection of TS analogs and intermediates in pharmaceutical wastewater and downstream aquatic environments has raised concern over the ecotoxicological effects of these substances and their contribution to feminization or masculinization in wildlife [55].

Despite these drawbacks, the pharmaceutical industry has historically favored and in many cases, still relies on chemical synthesis routes due to their proven scalability, relatively short production timelines, and well-characterized reaction mechanisms. For instance, the process disclosed in CN106397521A [38] enables high conversion efficiencies in the final transformation of AD to TS under optimized conditions, although such efficiencies are typically reported for specific experimental examples rather than as overall process yields and rely on stoichiometric chemical reduction rather than catalytic hydrogenation. Similarly, older patents such as US2742485A [38], despite being based on less environmentally conscious chemistries, illustrate the long-standing industrial reliance on reproducible, high-yielding chemical routes with predictable kinetics and well-defined product profiles.

In response to increasingly stringent environmental regulations and sustainability demands, several green chemistry principles have been progressively incorporated into steroid synthesis workflows. These efforts include the partial replacement of chlorinated solvents with alternatives such as ethyl acetate, the implementation of flow chemistry to improve operational safety and process control, and the development of solid-supported catalysts to facilitate catalyst recovery and reuse [56]. Nevertheless, such advances have not fully overcome the intrinsic limitations of multistep chemical steroid production, which continue to involve substantial solvent consumption, complex waste streams, and strict requirements for worker safety and regulatory compliance.

More recently, electrochemical methods have emerged as promising complementary approaches for selective steroid transformations. A notable academic example is the anodic oxidation strategy reported by Sommer et al. [57], which enables the efficient conversion of corticosteroids such as hydrocortisone or cortisone into valuable 17-ketosteroids through electrochemically driven C17 side-chain cleavage. This one-pot electrochemical procedure operates under relatively mild conditions using acetonitrile–water mixtures and carbon-based electrodes, achieving high selectivity and multigram scale product formation without the use of stoichiometric chemical oxidants.

Related electrochemical concepts have also been described in patent literature, including US3444057A [40] and WO2024261183A1 [41], which disclose electrochemical oxidation or reduction strategies applicable to steroidal substrates. However, these patents primarily address partial functionalization of steroid frameworks rather than complete TS synthesis, indicating that while electrochemical methods hold promise for specific redox transformations, their integration into fully industrialized TS production pipelines remains at an exploratory stage.

Nevertheless, although the electrochemical platform avoids the use of toxic oxidizing reagents and heavy metals, it is not entirely free from environmental and health concerns. The organic solvents employed, particularly acetonitrile (MeCN), are flammable, harmful upon inhalation, and classified as acutely toxic to aquatic life [58]. The supporting electrolytes typically used, such as tetraethylammonium tetrafluoroborate (Et_4_NBF_4_), are expensive, non-biodegradable, and may pose risks of bioaccumulation and ecotoxicity if not properly recovered or treated [57].

Although these salts can be recovered by evaporation and aqueous extraction after the reaction, such processes increase solvent consumption and energy use. Moreover, acetonitrile–water mixtures are not always efficiently separated at scale, which complicates solvent recycling [57].

From a process safety perspective, electrochemical setups operated under constant current can cause localized overheating and generate significant amounts of hydrogen gas at the cathode. If not properly vented, this hydrogen can accumulate and pose explosion risks, especially under scaled-up or continuous flow conditions [57,59].

Electrochemical oxidation of the C17 side-chain also generates glycolic acid, which can be further oxidized under the same conditions to form gaseous byproducts such as carbon dioxide and formaldehyde [57]. Although less toxic than chromium (VI) or selenium-based wastes, formaldehyde is a known carcinogen (IARC Group 1) and, even in small quantities, requires appropriate air management and gas scrubbing systems in enclosed reactor systems [60].

Taken together, all current routes to TS, including multistep chemical syntheses described in patents and innovative electrochemical platforms, differ in efficiency, atom economy, and scalability, but ultimately share the generation of environmentally hazardous effluents and potential health risks for workers and ecosystems. None of the existing approaches can be considered fully sustainable or benign.

Given these persistent challenges, there is growing industrial and academic interest in the development of biotechnological alternatives for TS synthesis. These approaches aim to reduce chemical waste, improve regio- and stereoselectivity, and align production practices with the principles of green chemistry, environmental safety, and the circular bioeconomy.

## 4. Biotechnological Production of Testosterone

Steroid molecules are structurally defined by a rigid tetracyclic fused ring nucleus and a functionalized side-chain at the C17 position, a molecular architecture that renders their complete chemical synthesis intrinsically complex and economically demanding [61].

Due to this structural complexity, pharmaceutical manufacturing strategies for steroid hormones have historically evolved toward combined chemical–biological approaches, in which microbial biotransformations are integrated with targeted chemical steps [62,63]. In this context, biotechnological processes have progressively assumed a central role in the early stages of steroid synthesis, particularly in the conversion of low-cost natural sterols into structurally simplified steroid intermediates suitable for further functionalization [64].

Traditionally, sapogenins, phytosterols, and cholesterol have been used as starting materials for industrial steroid synthesis [64], although current processes rely predominantly on phytosterols and cholesterol due to their lower cost, higher availability, and improved susceptibility to microbial transformation [65]. Among phytosterols, β-sitosterol, stigmasterol, and campesterol are the most abundant and industrially relevant substrates derived from plant biomass [66].

**Figure 2:**
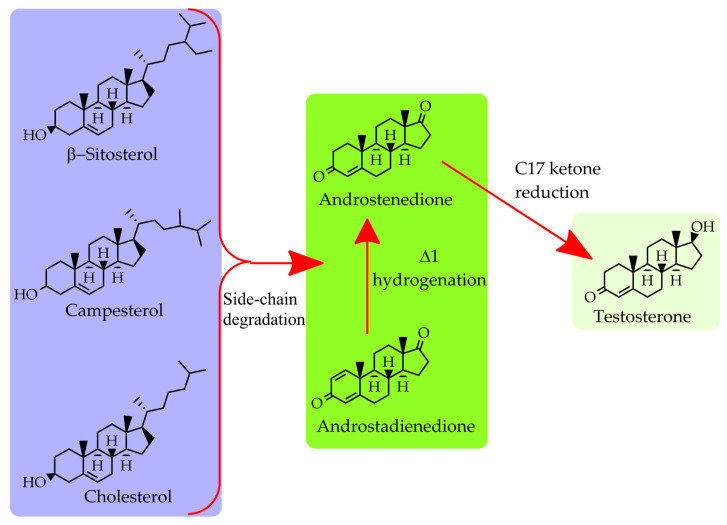
Simplified schematic representation of the key steroid transformations discussed in Section 4, including sterol side-chain cleavage to C19 intermediates (AD, ADD) and C17 ketone reduction to testosterone (TS).

The industrial synthesis of steroid hormones typically begins with the microbial biotransformation of phytosterols or cholesterol into key steroid intermediates through selective removal of the C17 side-chain. Complete side-chain cleavage yields C19 androstane derivatives, such as AD and 1,4-androstadiene-3,17-dione (ADD), which constitute the key intermediates for downstream androgen synthesis (Figure 2).

These transformations are predominantly carried out by actinobacteria of the genus *Mycolicibacterium* (formerly classified as *Mycobacterium*), particularly *M. smegmatis* and *M. neoaurum*, which possess specialized sterol catabolic pathways [67]. The microorganisms summarized in Table 4 illustrate the historical evolution from native sterol degrading strains to engineered *Mycolicibacterium* platforms optimized for selective accumulation of AD or ADD at industrially relevant substrate loadings.

Microbial degradation of sterol side-chains proceeds via β-oxidation–like mechanisms that efficiently remove the aliphatic substituent while preserving the steroid nucleus [62,76,83]. However, native sterol degrading microorganisms can also further metabolize the steroid core, leading to product losses. To overcome this limitation, industrial strains have been genetically optimized through targeted mutations in key catabolic enzymes, such as 3-ketosteroid-1-dehydrogenase (*KstD*) and 3-ketosteroid-9α-hydroxylase (*Ksh*). Disruption of these enzymes prevents degradation of the androstane nucleus and significantly enhances the accumulation of AD during fermentation [75,84].

The use of microbial biotransformations in steroid manufacturing dates back to the early twentieth century [65,85], culminating in the first industrial biotransformation patent for steroid hydroxylation. A landmark example is the Upjohn Company patent describing the microbial oxygenation of progesterone to 11α-hydroxyprogesterone using filamentous fungi (US2721163A) [86]. Since then, a wide range of microbial reactions, including hydroxylation, dehydrogenation, and reduction, have been incorporated into pharmaceutical steroid production processes [62,75].

From an industrial perspective, AD represents a central intermediate linking microbial sterol degradation with downstream androgen biosynthesis [64]. TS is an androstane derivative bearing a stereospecific 17β-hydroxyl group, which is responsible for its androgenic activity. While early studies reported direct TS formation by certain bacterial species, including *Mycobacterium* and *Lactobacillus* [62], industrial processes have largely converged on the use of AD as a universal precursor obtained via microbial transformation of sterols [65,87].

The conversion of AD into TS requires the stereospecific reduction of the C17 keto group to a 17β-hydroxyl moiety. This transformation can be achieved enzymatically through the action of 17β-hydroxysteroid dehydrogenases (17β-HSDs), enzymes that catalyze the NAD(P)H dependent reduction of 17-ketosteroids [87,88,89]. Once AD has been efficiently accumulated through microbial sterol biotransformation, the introduction of a stereospecific 17β-hydroxyl group becomes the defining step toward TS biosynthesis. This transformation represents a key technological decision point, as it can be accomplished via chemical reduction or enzyme catalyzed biotransformation, each associated with distinct implications for reaction selectivity, process integration, environmental sustainability, and downstream purification [90]. These enzymes have been identified in a variety of microbial and eukaryotic organisms and can be introduced into industrial strains through genetic engineering. Representative microbial and with enzymatic systems reported for this transformation are summarized in Table 5.

The table includes wild-type and engineered bacterial and fungal hosts, as well as cell free enzymatic systems catalyzing the stereospecific reduction of the C17 keto group via 17β-hydroxysteroid dehydrogenases or related reductases. Data were compiled from Sambyal and Singh (2020) [91] and updated with experimental studies published between 2021 and 2026.

Despite their industrial relevance, microbial steroid biotransformations are subject to several intrinsic biological and physicochemical limitations that directly impact process performance. Steroidal substrates are inherently hydrophobic and exhibit very low aqueous solubility, typically below the millimolar range, which restricts their bioavailability in fermentation media and can limit overall conversion efficiency [75]. Moreover, steroids exert toxic effects on many microorganisms at elevated concentrations, leading to growth inhibition and reduced biocatalytic activity, particularly under high substrate loading conditions [65]. As a result, both substrate loading and microbial tolerance remain key parameters that must be carefully balanced during process optimization [62].

To address these challenges, multiple process level strategies have been developed to enhance steroid bioavailability and microbial tolerance. These include substrate micronization, the use of organic aqueous biphasic or microemulsion systems, and the incorporation of hydrophobic carriers or oils that act as reservoirs for sterol substrates and products, thereby reducing local toxicity and improving mass transfer [65,75]. In parallel, industrial production strains have been progressively adapted or engineered to withstand higher steroid concentrations, further improving robustness and productivity compared to wild-type microorganisms [65].

Advances in metabolic engineering have demonstrated the feasibility of constructing complex steroidogenic pathways in microbial hosts. Notably, the complete biosynthesis of cortisone has been achieved in yeast through the coordinated expression of thirteen heterologous genes, illustrating the potential of synthetic biology to replace multistep chemical synthesis with integrated biological routes [100]. Although current biotechnological processes for TS production generally yield lower volumetric productivity than fully chemical routes, continuous advances in enzyme engineering, pathway optimization, and process intensification have progressively reduced this gap, particularly at the pilot and demonstration scales [88,89,95].

Taken together, microbial biotransformation strategies have become an indispensable component of modern TS manufacturing. By providing efficient access to key steroid intermediates and enabling highly selective functional group modifications, biotechnological processes complement and progressively reshape conventional chemical synthesis routes. These characteristics establish a framework for directly comparing biological and chemical approaches in terms of efficiency, selectivity, and environmental performance.

## 5. Environmental and Sustainability Considerations

The industrial production of TS has raised significant environmental concerns, particularly when conventional chemical synthesis routes are employed. These processes are commonly characterized by high energy demand, extensive use of hazardous solvents and reagents, and the generation of toxic and persistent waste streams, including heavy metals, carcinogenic byproducts, and endocrine disrupting compounds [48,101]. Such features have increasingly drawn regulatory scrutiny due to their potential ecological and human health impacts [102].

Traditional chemical routes for TS synthesis typically involve 10 to 15 reaction steps, with overall conversion efficiencies rarely exceeding 75%. This multistep nature leads to the accumulation of complex effluents that are difficult to treat and may pose significant ecotoxicological risks if discharged without adequate remediation [103,104]. Although recent innovations, including electrochemical steroid transformations, aim to reduce the reliance on highly toxic oxidants, these approaches still depend on organic solvents such as acetonitrile and non-biodegradable supporting electrolytes, which may persist in aquatic environments and contribute to long term ecological burdens [105,106].

In contrast, biotechnological strategies based on microbial biotransformations have been increasingly explored as environmentally favorable alternatives aligned with green chemistry principles and emerging international sustainability frameworks [87,89,107]. These processes typically operate under milder conditions, including aqueous media, ambient temperatures, and atmospheric pressure, and exhibit high regio- and stereoselectivity. As a consequence, biotechnological routes are generally associated with lower energy consumption, reduced waste generation, and simplified downstream processing compared to conventional chemical synthesis [108,109].

An additional advantage of microbial TS production lies in its compatibility with renewable and circular feedstocks. In particular, phytosterols recovered from agroindustry byproducts can serve as effective carbon sources, supporting circular bioeconomy models and improving overall resource efficiency [93,110]. These features contribute to a reduction in the environmental footprint associated with raw material sourcing and upstream processing.

Life cycle assessment (LCA) studies comparing chemical and biotechnological routes for steroid and pharmaceutical compound production have reported substantial environmental benefits associated with microbial systems [111,112]. Under optimized fermentation and recovery conditions, reductions of up to 40–60% in greenhouse gas emissions and more than 70% in ecotoxicity indicators have been described relative to conventional chemical synthesis [113]. However, these advantages must be balanced against additional environmental burdens associated with large-scale fermentation processes, including increased resource demand and energy requirements related to bioreactor operation [114,115].

A comparative overview of carbon footprint, energy demand, waste generation, and ecotoxicity associated with chemical and biotechnological TS production routes is summarized in Table 6. While biotechnological processes generally exhibit a more favorable environmental profile, continued process optimization remains essential to minimize water and energy inputs. Proposed mitigation strategies include nutrient recycling, the use of low impact nitrogen sources, closed loop water management systems, and improved bioreactor design to enhance mass transfer efficiency [116,117].

Although phytosterols used as starting materials may originate from renewable biomass, conventional chemical synthesis routes rely extensively on petrochemical-derived solvents, stoichiometric reagents, and non-renewable catalytic systems. In this context, the expression “limited compatibility with renewable carbon sources” refers not to the renewable origin of sterol substrates themselves, but to the absence of systemic integration of chemical processes with renewable carbon fluxes, circular feedstocks, and metabolically adaptable production platforms. In contrast, microbial systems can directly utilize renewable substrates within integrated metabolic networks, thereby enabling improved carbon circularity and process sustainability [111,113].

The comparison is based on reported life cycle assessment (LCA) studies of steroid synthesis and analogous pharmaceutical manufacturing processes. Values are presented qualitatively or as relative trends, as direct industrial LCA data for TS production remain limited.

In parallel, policy initiatives such as the European Union’s Pharmaceutical Strategy increasingly promote the adoption of cleaner production routes through fiscal incentives, environmental performance scoring, and expedited regulatory pathways for sustainable pharmaceutical manufacturing [138,139]. Within this regulatory and technological landscape, biotechnological TS production emerges as a promising approach to reduce the environmental impact of steroid manufacturing, provided that microbial platforms, downstream processing, and life cycle aware process integration continue to advance in a coordinated manner.

## 6. Future Perspectives and Industrial Challenges

Despite the considerable environmental advantages offered by biotechnological TS production, numerous scientific and industrial barriers must still be overcome to enable large-scale deployment [95]. Among the highest priorities are the enhancement of microbial platforms for steroid biotransformation, the stabilization of engineered pathways, and the development of scalable and economically competitive production systems [75,140,141]. In this context, both strain level optimization and process level innovation must progress in parallel to facilitate industrial translation [142].

Extensive efforts have been focused on the genetic engineering of *Mycolicibacterium* and *Rhodococcus* strains, which possess natural catabolic pathways for sterol degradation [143]. In these actinobacteria, the heterologous or amplified expression of 17β-hydroxysteroid dehydrogenases (17β-HSDs) has enabled efficient conversion of AD into TS, completing the final and critical reductive step in androgen biosynthesis [143]. For instance, engineered *Mycolicibacterium neoaurum* strains harboring 17β-HSD from *Comamonas testosteroni* have achieved TS titers above 400 mg/L under fed-batch fermentation, with conversion efficiencies exceeding 85% from AD [87].

Beyond dehydrogenase-based systems, cytochrome P450 monooxygenases (CYP450s), particularly those belonging to the CYP17 and CYP68 families, have received growing attention for their ability to catalyze regio- and stereoselective hydroxylation reactions that are difficult to reproduce through chemical synthesis [144,145]. Through protein engineering and directed evolution, substrate specificity, turnover rates, and resistance to steroidal toxicity have been enhanced in these oxygenases, expanding their applicability in microbial steroid biosynthesis [146,147].

Fungal systems have also emerged as promising hosts for steroid biosynthesis, owing to their eukaryotic nature, genetic tractability, and scalability [148,149]. Recent studies have demonstrated that *Aspergillus nidulans*, a widely used industrial microorganism, can be engineered to produce TS from AD by heterologous expression of bacterial 17β-HSD genes. In this system, conversion yields approaching 70% were achieved under optimized fermentation conditions, representing one of the few reported examples of direct TS biosynthesis in a filamentous fungal host [89]. The use of *A. nidulans* as a cell factory offers advantages in terms of safety, robustness, and compatibility with existing industrial fermentation infrastructure.

Furthermore, *Aspergillus ochraceus* has been shown to harbor CYP68L8, a cytochrome P450 monooxygenase capable of catalyzing 11α-hydroxylation of AD and progesterone with high specificity and efficiency [89]. Heterologous expression of CYP68L8 in *A. nidulans* restored steroid hydroxylation activity in CYP deficient mutants, confirming its biocatalytic functionality [89]. Given the historical use of *A. ochraceus* in large-scale fungal fermentations, the removal of endogenous CYP activity with heterologous 17β-HSD expression may enable the construction of robust fungal platforms for complete TS biosynthesis from sterol precursors [150,151]. The sporulation capacity, environmental resilience, and compatibility with low sterility or non-sterile processes further enhance the industrial potential of these species.

Together, these examples highlight filamentous fungi not merely as auxiliary biocatalysts, but as emerging eukaryotic production platforms capable of integrating oxidative and reductive steroid transformations within a single host [152]. Their genetic tractability, tolerance to hydrophobic substrates, and compatibility with large-scale fermentation infrastructure position filamentous fungi as promising platforms for consolidated bioprocesses in industrial TS production [153].

In parallel with advances in microbial chassis engineering, process intensification strategies are expected to play a decisive role in accelerating the industrialization of microbial TS production. The implementation of continuous and semi-continuous bioprocesses has been widely recognized as an effective approach to increase space time yields, improve process stability, and reduce downtime associated with batch operations [154,155]. In addition, co-culture systems, combining sterol degrading microorganisms with specialized steroid transforming strains, may enable pathway modularization and improved metabolic efficiency by distributing biosynthetic functions across complementary microbial partners [75,156].

Immobilized cell and biofilm based catalytic systems represent another promising technological route, offering enhanced operational stability, improved tolerance to steroidal toxicity, and the possibility of catalyst reuse over extended production cycles [75,157]. Such systems have been successfully applied in industrial biotransformations involving redox intensive reactions, where cofactor regeneration and mass transfer limitations can be mitigated through spatial organization of the biocatalyst [158,159]. These features are particularly relevant for AD reduction and cytochrome P450 mediated hydroxylation reactions [75].

Moreover, the intrinsic robustness of several actinobacterial and filamentous fungal hosts supports the development of non-sterile or low sterility fermentation strategies, which have been shown to substantially reduce capital and operational costs while maintaining process productivity in large-scale industrial settings [160,161]. Such approaches are desirable for bulk steroid intermediates, where stringent sterility requirements represent a major economic constraint.

Pilot-scale fed-batch bioreactor studies using engineered *Mycolicibacterium neoaurum* strains have demonstrated TS production from phytosterols, achieving titers of up to approximately 4.6 g/L under optimized conditions [95]. These pilot studies demonstrate the scalability and robustness of bacterial systems, as well as the feasibility of integrating upstream biotransformation with downstream crystallization and purification.

Nevertheless, beyond technological development, regulatory acceptance of genetically modified microorganisms (GMOs) for pharmaceutical applications remains a critical constraint [162]. Industrial-scale processes must comply with biosafety, quality assurance (GMP), and environmental impact regulations, as well as intellectual property and data protection frameworks [163]. In addition, the economic viability of microbial TS production depends on minimizing feedstock costs, adapting existing reactor infrastructure, and optimizing catalyst reuse and process continuity to reduce overall operational expenditures [154,164].

It is expected that continuing advances in synthetic biology, such as modular metabolic engineering, genome scale pathway modeling, and CRISPR/Cas based gene regulation, will further accelerate the development of highly efficient microbial chassis tailored for steroid biosynthesis [155,165]. The convergence of these molecular tools with advanced bioprocess schemes, including continuous operation, co-culture design, immobilized biocatalysts, and non-sterile fermentation, is likely to be critical for achieving economically viable and environmentally competitive TS biomanufacturing [166,167].

From a molecular perspective, future progress in TS biomanufacturing will largely depend on the rational engineering of key steroid transforming enzymes, the stabilization of engineered metabolic nodes, and the coordinated regulation of sterol uptake, cofactor balance, and product export within microbial hosts [143,168].

## 7. Conclusions

The industrial production of TS has traditionally relied on chemical synthesis due to its efficiency, scalability, and well-established technological foundation. However, this approach faces growing environmental, regulatory, and societal pressures resulting from the use of hazardous reagents, high energy consumption, and the generation of persistent and toxic waste streams. These limitations underscore the urgent need to explore more sustainable alternatives.

Biotechnological methods, particularly those based on microbial biotransformation and synthetic biology, have emerged as promising alternatives, offering significant advantages in terms of environmental sustainability, reaction specificity, and alignment with circular economy principles. While microbial platforms are still undergoing optimization for industrial deployment, recent advances in strain engineering, metabolic pathway design, and integrated bioprocessing have demonstrated their potential to meet industrial performance criteria.

The future of TS manufacturing will depend on the successful integration of biotechnological platforms into real world production environments. This includes not only improving biocatalyst productivity and process robustness but also navigating regulatory pathways and ensuring economic viability at commercial scale. As global pharmaceutical industries move toward greener manufacturing standards, the transition to sustainable steroid production represents not only an environmental necessity but also a strategic opportunity for innovation, competitiveness, and leadership within the emerging bioeconomy.

## Figures and Tables

**Table 1:** Global TS market size and projected growth trends (data from [25]).

Year	Total Market (USD Millions)	The Compound Annual Growth Rate (CAGR) (%)
2023	2550	-
2025	2850	5.8
2030	3400	5.5
2032	3520	5.2

**Table 2:** Distribution of TS products by route of administration (adapted from [26]).

Method of Introducing a Drug	Market Share (%)
Injectables	45
Transdermal	30
Oral	15
Subcutaneous	10

**Table 3:** Representative patented chemical routes for industrial TS synthesis.

Starting Material	Main Intermediates	Number of Steps	Key Reagents/ Solvents	Overall Yield (%)	Main Environmental and Safety Concerns	Patent/ Reference
4-androstene-3,17-dione	Androstenedione-17-monocyanohydrin; enol ethers/ketals	4–6	Acetone, cyanohydrin, ethyl orthoformate, sodium, benzene, pyridine	~85–90	Cyanide toxicity, benzene use, alkali metals, and multistep waste generation	[37]
4-androstene-3,17-dione	Enol ether/oxime derivatives; 17β-hydroxy intermediates	3	Ethyl orthoformate, hydroxylamine, NaBH_4_/KBH_4_, alcohol solvents, mineral acids	~70–75	High solvent consumption, borohydride reagents, acid–base waste streams, multistep purification	[38]
4-androstene-3,17-dione	17-keto-3-enol ether; 3-enol ether intermediates	5–7	Triethyl orthoformate, NaBH_4_/LiBH_4_, mineral acids, THF, cyclohexane, alcohols	Overall ~70–80% estimated	High solvent use, borohydride reagents, protection/deprotection steps, multistep purification	[36]
Aromatic steroid derivatives	Electroreduced steroid nucleus. Not direct to TS	1–2 (electrochemical)	Electrolysis cell, aprotic solvents, supporting electrolyte	Not stated	Electrolyte recovery, energy input, and solvent toxicity	[39]
Cholesterol/phytosterols	Allylic C–H oxidation of sterol skeleton	1 (electrochemical)	Electrochemical oxidation, organic solvent, supporting electrolyte	Not stated	Electrolyte recovery, solvent use, and anodic oxidation byproducts	[40]
Cholesterol/phytosterols	Oxidized sterol intermediates; C19 androstane derivatives	>10	Strong oxidants, acids, metal reagents, and organic solvents	Not stated	Multistep synthesis, hazardous reagents, poor atom economy	[41]
Phytosterols	Androstane intermediates leading to AD	>10	Acidic and basic treatments, metal mediated reductions, and solvents	Not stated	Low overall efficiency, solvent waste, and harsh conditions	[42,43]

**Table 4:** Microorganisms are reported to biotransform sterols into C19 steroid intermediates, including androstenedione (AD) and 1,4-androstadiene-3,17-dione (ADD). Molar yields are reported only when explicitly defined in the original publications; n.r. indicates values that were not reported. The microorganisms listed encompass the strains compiled in the reviews by Malaviya and Gomes (2008) and by Nunes (2022) [64,65], together with subsequent developments reported in the literature and selected industrial patents.

Microorganism	Substrate (g·L^−1^)	Main Product	Molar Yield (%)	Reference
*Mycobacterium* sp. NRRL B-3805	Lanosta-7,9(11)-dien-3β-ol (0.25)	4,8(14)-Androstadiene-3,17-dione	30	[67]
*Moraxella* sp.	3β-Acetoxy-19-hydroxycholest-5-ene (0.5)	Estrone	15	[68]
*Mycobacterium* sp. NRRL B-3805	Ergosterol (0.3)	AD	35	[69]
*Mycobacterium* sp. NRRL B-3683	Ergosterol (0.3)	ADD	30	[69]
*Mycobacterium* sp. NRRL B-3805	α-Sitosterol (1.0)	AD	20–25	[70]
*Mycobacterium* sp. NRRL B-3805	β-Sitosterol (1.0)	AD	90	[71]
*Mycobacterium* sp. VKM Ac-1815D ET1	β-Sitosterol (5.0)	AD	72	[72]
*Mycobacterium* sp. MB-3683	Phytosterols (10)	AD	90	[73]
*Mycobacterium* sp. MB-3683	Phytosterols (30)	AD	80	[73]
*Mycolicibacterium neoaurum*	Phytosterols	AD	>90	[63,74,75]
*Mycolicibacterium neoaurum* TCCC 11978	Phytosterols (3)	AD	55.8	[76]
*Mycolicibacterium neoaurum* NwIB-R10hsd4A	Phytosterols	AD/ADD	24.7 g·L^−1^ (resting cells)	[77]
*Mycolicibacterium neoaurum* (ΔkstD/Δksh mutants)	Phytosterols	AD	>90	[63]
*Rhodococcus erythropolis*	Phytosterols (20–30)	AD/9-OH-AD	~65	[64]
*Gordonia* sp.	Cholesterol	AD/ADD	87.2	[78]
*Moraxella ovis*	Rice bran oil (RBO)	AD/ADD	0.22 mg AD/40 mg RBO	[79]
*Pseudomonas* sp. NCIB 10590	β-Sitosterol	AD	n.r. ^1^	[80]
*Mycobacterium vaccae*	β-Sitosterol	AD	n.r. ^1^	[81]
*Mycobacterium fortuitum*	β-Sitosterol	AD/ADD	n.r. ^1^	[82]

^1^ n.r. indicates values that were not reported.

**Table 5:** Microorganisms and biocatalytic systems have been reported to convert sterols or AD into TS.

Microorganism	Substrate	Main Product	Molar Yield (%)	Reference
*Mycobacterium* sp.	Cholesterol	TS	~51% molar conversion	[87]
*Mycobacterium* sp. MB-3638	Cholesterol	TS	~98% conversion (72 h)	[87]
*Mycobacterium* sp. NRRL B-3683 mutant	AD	TS	High conversion (qualitative)	[91]
Engineered *Mycobacterium smegmatis* (heterologous 17β-HSD)	AD/sterols	TS	~80% conversion	[86]
ST2 mutant derived from *Mycobacterium* sp. B-3805S	Phytosterols → AD	TS	~31% overall phytosterol → TS	[92]
*Mycobacterium* mutant VKM Ac-1816D (high 17β-HSD activity)	β-Sitosterol	TS	~50–55% molar yield	[93]
*Mycolicibacterium neoaurum* VKM Ac-1815D (one-pot mode)	Phytosterols (10 g·L^−1^)	TS	4.59 g·L^−1^ (~66% molar yield)	[94]
*Mycolicibacterium neoaurum* VKM Ac-1816D (oxidative/reductive modes)	Phytosterols (5 g·L^−1^)	TS	1.83 g·L^−1^ (~52.5% yield)	[94]
*Lactobacillus bulgaricus*	Cholesterol/AD	TS	1.56 mmol·L^−1^ (96 h)	[95]
*Saccharomyces cerevisiae* (biotransformation system)	AD	TS	Efficient reduction (cyclodextrin assisted)	[96]
*Aspergillus terreus* PTCC 5283	AD	TS, testololactone	Qualitative TS formation	[97]
Engineered *Aspergillus nidulans* (heterologous 17β-HSD)	AD	TS	~70% conversion under optimized conditions	[31]
Zoosporic fungi	Progesterone	TS, testololactone	Qualitative	[98,99]

**Table 6:** Comparative environmental performance of chemical and biotechnological TS production routes.

Parameter	Chemical Synthesis Routes	Biotechnological Routes
Carbon footprint (GHG emissions)	High, due to multistep synthesis, elevated temperatures, pressure requirements, and extensive use of fossil derived reagents and organic solvents; life cycle assessment (LCA) studies report substantial CO_2_ equivalent emissions associated with solvent production, energy demand, and waste treatment [117,118].	Moderate to low; LCA studies on microbial steroid biotransformations and related pharmaceutical bioprocesses report up to 40–60% reduction in greenhouse gas emissions under optimized fermentation and downstream processing conditions [119,120].
Energy consumption	High, driven by repeated heating, cooling, solvent recovery, distillation, and purification steps across 10–15 reactions [121].	Moderate; processes typically operate at ambient temperature and pressure, with energy demand mainly associated with aeration, agitation, and downstream recovery [122,123].
Hazardous reagents	Extensive use of toxic oxidants, heavy metals, chlorinated or aromatic solvents, and non-renewable reagents, generating hazardous waste streams [124,125].	Substantially reduced; predominantly aqueous media, biocatalysts, and biodegradable nutrients, with limited reliance on hazardous chemicals [120].
Waste generation	High volumes of hazardous and persistent waste streams, including metal containing residues, spent solvents, and endocrine disrupting byproducts, require complex treatment and disposal strategies [126].	Lower overall waste generation; waste streams mainly consist of microbial biomass, spent culture media, and biodegradable residues [127].
Ecotoxicity potential	High; untreated or insufficiently treated effluents from chemical synthesis may pose significant ecotoxicological risks due to persistent organic pollutants and heavy metals [128].	Significantly reduced; LCA studies report up to ~70% lower ecotoxicity burden for microbial processes compared to chemical routes when optimized waste management and recovery strategies are applied [129].
Water consumption	Moderate; primarily associated with solvent washing, extraction, and purification steps [130,131].	High; substantial water input required for fermentation media preparation, cleaning in place operations, and downstream processing [132].
Process integration with renewable feedstocks	Limited systemic integration; although sterol substrates may be renewable, the process remains highly dependent on petrochemical reagents and solvents [111,133].	High; compatible with phytosterols recovered from agroindustry byproducts and circular bioeconomy models, improving resource efficiency [109,134].
Regulatory and environmental compliance	Increasingly constrained by stringent environmental regulations, waste management costs, and restrictions on hazardous substances [135,136].	Better alignment with green chemistry principles and emerging sustainability policies, facilitating regulatory approval and incentive-based adoption [118,137].

## Data Availability

No new data were created or analyzed in this study. Data sharing is not applicable to this article.
